# A pilot study on interobserver variability in organ-at-risk contours in magnetic resonance imaging-guided online adaptive radiotherapy for pancreatic cancer

**DOI:** 10.3389/fonc.2024.1335623

**Published:** 2024-05-10

**Authors:** Marie Kurokawa, Masato Tsuneda, Kota Abe, Yohei Ikeda, Aki Kanazawa, Makoto Saito, Asuka Kodate, Rintaro Harada, Hajime Yokota, Miho Watanabe, Takashi Uno

**Affiliations:** ^1^ Diagnostic Radiology and Radiation Oncology, Graduate School of Medicine, Chiba University, Chuo-ku, Chiba, Japan; ^2^ Department of Radiation Oncology, MR Linac ART Division, Graduate School of Medicine, Chiba University, Chuo-ku, Chiba, Japan; ^3^ Department of Radiology, Chiba University Hospital, Chuo-ku, Chiba, Japan

**Keywords:** MRgOART, pancreatic cancer, organs-at-risk, butylscopolamine, contouring, interobserver variability

## Abstract

**Purpose:**

Differences in the contours created during magnetic resonance imaging-guided online adaptive radiotherapy (MRgOART) affect dose distribution. This study evaluated the interobserver error in delineating the organs at risk (OARs) in patients with pancreatic cancer treated with MRgOART. Moreover, we explored the effectiveness of drugs that could suppress peristalsis in restraining intra-fractional motion by evaluating OAR visualization in multiple patients.

**Methods:**

This study enrolled three patients who underwent MRgOART for pancreatic cancer. The study cohort was classified into three conditions based on the MRI sequence and butylscopolamine administration (Buscopan): 1, T2 imaging without butylscopolamine administration; 2, T2 imaging with butylscopolamine administration; and 3, multi-contrast imaging with butylscopolamine administration. Four blinded observers visualized the OARs (stomach, duodenum, small intestine, and large intestine) on MR images acquired during the initial and final MRgOART sessions. The contour was delineated on a slice area of ±2 cm surrounding the planning target volume. The dice similarity coefficient (DSC) was used to evaluate the contour. Moreover, the OARs were visualized on both MR images acquired before and after the contour delineation process during MRgOART to evaluate whether peristalsis could be suppressed. The DSC was calculated for each OAR.

**Results:**

Interobserver errors in the OARs (stomach, duodenum, small intestine, large intestine) for the three conditions were 0.636, 0.418, 0.676, and 0.806; 0.725, 0.635, 0.762, and 0.821; and 0.841, 0.677, 0.762, and 0.807, respectively. The DSC was higher in all conditions with butylscopolamine administration compared with those without it, except for the stomach in condition 2, as observed in the last session of MR image. The DSCs for OARs (stomach, duodenum, small intestine, large intestine) extracted before and after contouring were 0.86, 0.78, 0.88, and 0.87; 0.97, 0.94, 0.90, and 0.94; and 0.94, 0.86, 0.89, and 0.91 for conditions 1, 2, and 3, respectively.

**Conclusion:**

Butylscopolamine effectively reduced interobserver error and intra-fractional motion during the MRgOART treatment.

## 1 Introduction

In patients with unresectable locally advanced pancreatic cancer, conventional radiation therapy confers a slight survival advantage compared with chemotherapy alone, necessitating the discovery of more effective local approaches (1–3). Dose escalation is essential to achieve local tumor control and improve overall survival. However, dose cannot be escalated due to restrictions on the tolerable dose of healthy organs surrounding the pancreas. Advances in irradiation techniques, such as intensity-modulated radiation therapy, facilitate the administration of high doses while minimizing the dose to the organs at risk (OAR). Treatment of unresectable pancreatic cancer using a general linear accelerator is performed in 15 or 25 fractions, while meeting the dose limit to the surrounding normal organs, using countermeasures against respiratory migration and image-guided technology (4, 5). Recently, magnetic resonance imaging-guided Online Adaptive Radiation Therapy (MRgOART), which fully uses MR image-guided and online adaptive technology for pancreatic cancer, has enabled more effective dose prescription (6–9).

Recommendations for precise delineation of tumors and OARs in abdominal regions with respiratory movements and intestinal peristalsis have been reported (10). Motion artifacts occur due to the movement of internal organs during MRI, reducing visibility. Mostafaei et al. investigated body movements induced by breathing and peristalsis on computed tomography (CT) and MRI scans acquired during free breathing and breath-holding. They concluded that evaluating both respiratory movement and peristalsis is essential (11). Breath-holding and abdominal compression have been reported as countermeasures for respiratory movement (7–9). The pre-treatment images can directly correct the current gastrointestinal position and movement in an online adaptive radiotherapy plan. Some drugs can also inhibit peristalsis (12). No study has investigated whether it is possible to accurately defininig the contours of normal organs on images affected by motion artifacts could be possible.

This study aimed to evaluate interobserver error while delineating OARs in patients with pancreatic cancer treated with MRgOART. Furthermore, we also aimed to demonstrate the utility of drugs that suppress peristalsis by evaluating OAR visualization in multiple patients.

## 2 Methods

### 2.1 Patient data and MRI

**Figure 1:**
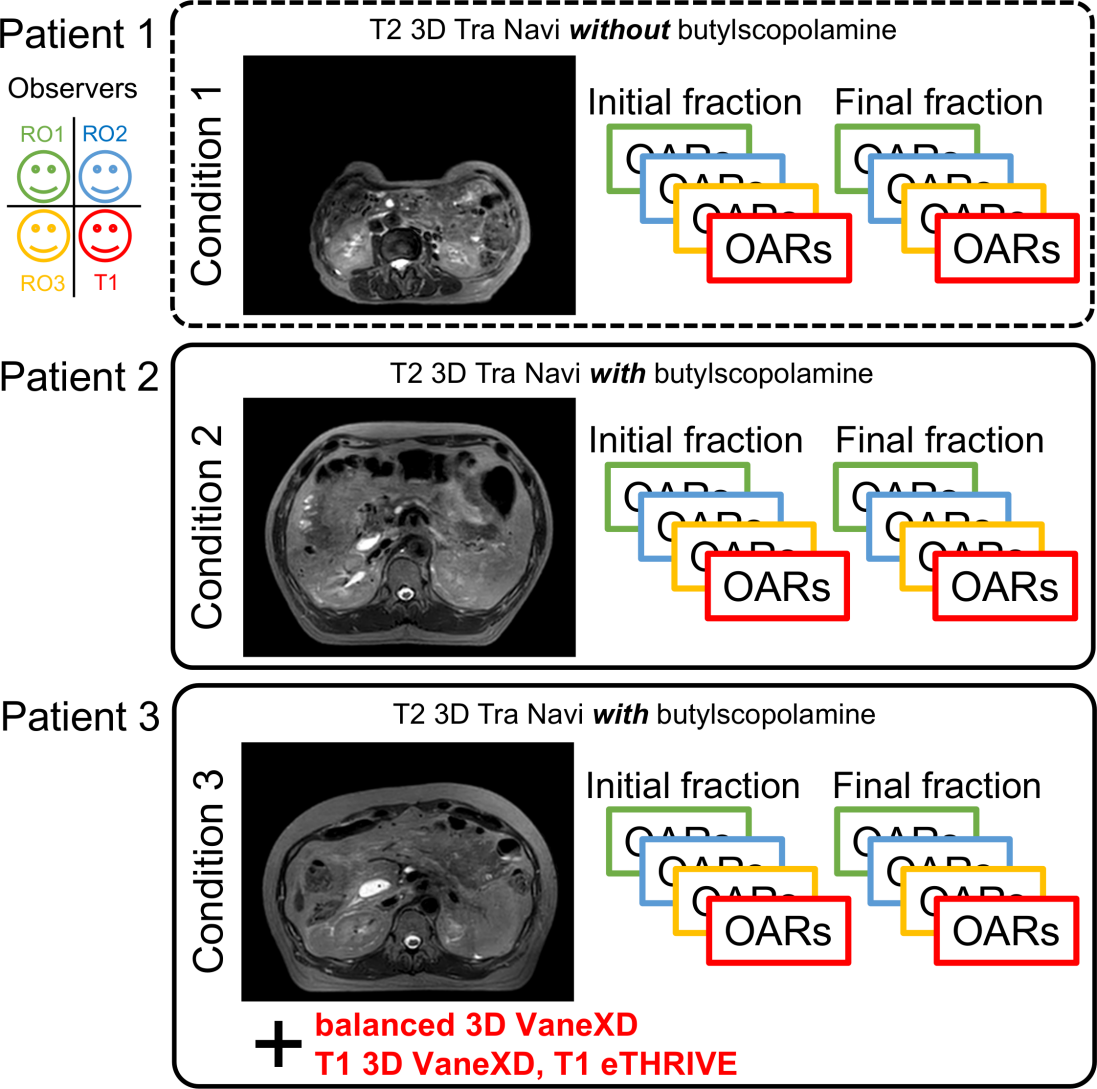
Magnetic resonance imaging (T2 3D Tra Navi image) and schematic representation of our delineation study.

This study was approved by the Ethics Committee of Chiba University Hospital (HK202304-07). The study enrolled three patients who underwent MRgOART for pancreatic cancer using Elekta Unity MR-linac (Elekta, Stockholm, Sweden). The patient details are presented in **Table 1**. At our hospital, MRIs are performed using a T2 navigator echo sequence under abdominal compression to measure respiratory movement. Patients 2 and 3 (conditions 2 and 3) who could be treated with butylscopolamine bromide (Buscopan®Injection, Paris, France) were administered the drug to suppress intestinal peristalsis. Butylscopolamine was deemed contraindicated in Patient 1 (condition 1) owing to a history of valvular heart disease. Moreover, our hospital incorporates contour delineation on images captured in multiple sequences into the workflow using the treatment planning support device MIM Maestro (MIM Software, 7.1.5, Cleveland, OH, USA) (condition 3) (9, 13). The imaging sequences provided by the vendor were used for MRI acquisition, except for one sequence, T1-eTHRIVE. **Table 2** presents the parameters of the imaging sequence. **Figure 1** shows an example of a treatment image captured using the T2 3D Tra Navi sequence and provides an overview of our delineation study.

**Table 1:** Patient overview.

Patient No.	I	II	III
Condition No.	1	2	3
Age (years)	78	59	51
Sex	F	M	M
Tumor location	Head of the pancreas	Uncinate process of the pancreas	Head of the pancreas
Prior chemotherapy	GEM+nabPTX	GEM+nabPTX→mFOLFIRINOX	GEM+nabPTX→mFOLFIRINOX
KPS at the start of ablative radiotherapy	90	90	100
Imaging sequence	T2 3D Tra Navi	T2 3D Tra Navi	T2 3D Tra Navi with optional imaging
Abdominal compression	+	+	+
Butylscopolamine (20 mg/ampule)	−	+	+

GEM, gemcitabine; nabPTX, nanoparticle albumin-bound paclitaxel; FOLFIRINOX, oxaliplatin, irinotecan, fluorouracil, and leucovorin; KPS, Karnofsky Performance Status.

**Table 2:** Overview of imaging sequences.

Name	T2 3D Tra Navi	b3D VaneXD	T1 3D VaneXD	eTHRIVE*
Scan technique	T2-TSE	B-FFE	T1-FFE	T1-FFE
Scan time	2:21	6:51	6:01	2:29
Voxel size	0.79 × 0.79 × 1.2	0.78 × 0.78 × 1.5	0.78 × 0.78 × 1.5	0.78 × 0.78 × 2.4
Field-of-view	360 × 455	500 × 500	500 × 500	360 × 438
TR/TE	2100/102	3.3/1.31	3.9/1.18	4.6/2.3
ETL	167	94	−	15
FA	90	40	15	10
NEX	2	1	1	5
Fat suppression	−	−	−	+

*These sequences are developed at our facility.

TR/TE, repetition time/echo time; ETL, echo train length; NEX, number of excitations; FA, flip angle.

### 2.2 Evaluation of contouring

The observers were three radiation oncologists (RO1, RO2, and RO3) and one resident physician (T1); the other observers were blinded to the contours during contour delineation. RO1, RO2, RO3, and T1 had treatment experiences of 14, 15, 13, and 3 years, respectively. In this study, MR images from the initial and final MRgOART sessions were used to delineate the contours of OAR for each condition. Each observer created an outline of the stomach, duodenum, small intestine, and large intestine on this MR image. The range for delineating the contour was an area extending ±2 cm in the craniocaudal direction from the slice coordinates of the planning target volume (PTV). The primary outcome measure was the agreement between observers for each contour. The dice similarity coefficient (DSC) was used to assess interobserver agreement for each contour in each patient (14). DSC has been used broadly in the field of segmentation as a measure of spatial overlap ranging between 0 and 1, where 0 indicates no overlap and 1 indicates exact overlap. The OARs were depicted using the MIM software and exported after anonymization. DSC values were calculated using an in-house program developed in Python and subsequently averaged.

### 2.3 Peristaltic motion

Two MR images obtained before and after contouring were used to evaluate intestinal peristalsis. In the MRgOART workflow, MR images (pre-treatment MR images) were acquired before the treatment planning. The contouring, optimization, and dose calculation were performed immediately using this image. Since this process was time-consuming, position verification MR images could be acquired immediately before irradiation to assess patient misalignment (15, 16). These two images were compared, and the process proceeded to irradiation if no positional shift was found. The contours to be evaluated were the duodenum, stomach, small intestine, and large intestine, as described above. One observer (RO1) compared the contours using the DSC.

### 2.4 Evaluation of contouring (distance and area of overlapping)

**Figure 2:**
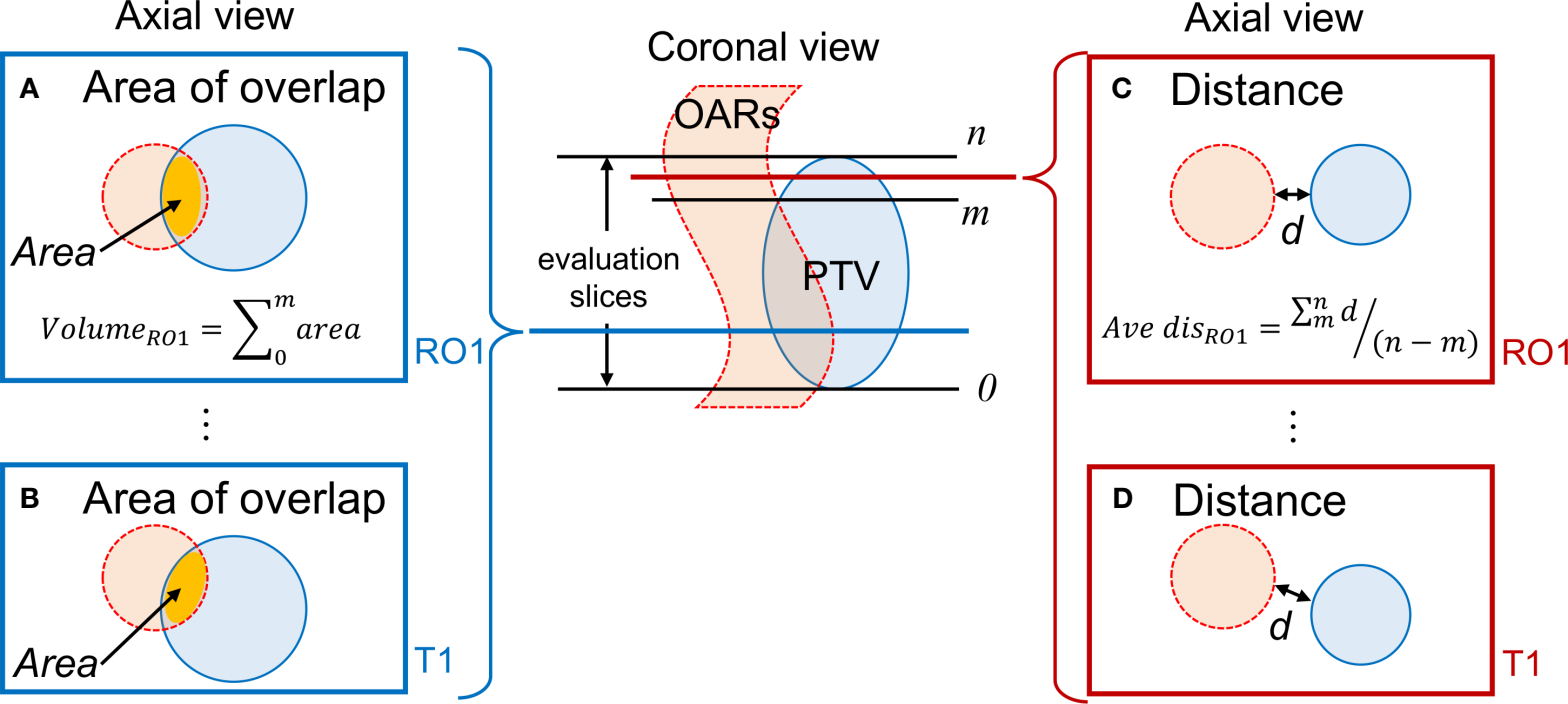
Evaluation of the positional relationship between the PTV and OARs. **(A, B)** Calculation of the overlap area for RO1 and T1 when the PTV and OARs overlap; **(C, D)** Determination of the shortest distance *d* for RO1 and T1 when there is no overlap between the PTV and OAR. Evaluation is conducted on slices containing the PTV. PTV, planning target volume; OAR, organ-at-risk.

**Figure 2** depicts a conceptual diagram of the distance and area of overlap between the PTV and OARs. We determined the presence or absence of overlap between the PTV and OAR in each slice containing the PTV (range, 0–n). Regarding overlapping region for each observer, the area of overlap was calculated and accumulated, representing the overlap volume. In cases wherein no overlap occurred, the shortest distance between the PTV and each OAR contour was calculated. The average value of the shortest distances for each observer was evaluated. MR images of two sessions were analyzed. These indicators were calculated using an in-house program developed in Python. In all OARs, the values of overlap volume and averaged shortest distance were calculated for each combination of condition and observers. The values for all observers were averaged. We calculated the standard deviation (SD) and coefficient of variation (CV) as a measure of interobserver error. Larger CVs indicated greater interobserver differences among observers in these indicators.

## 3 Results

### 3.1 Treatment time

**Figure 3:**
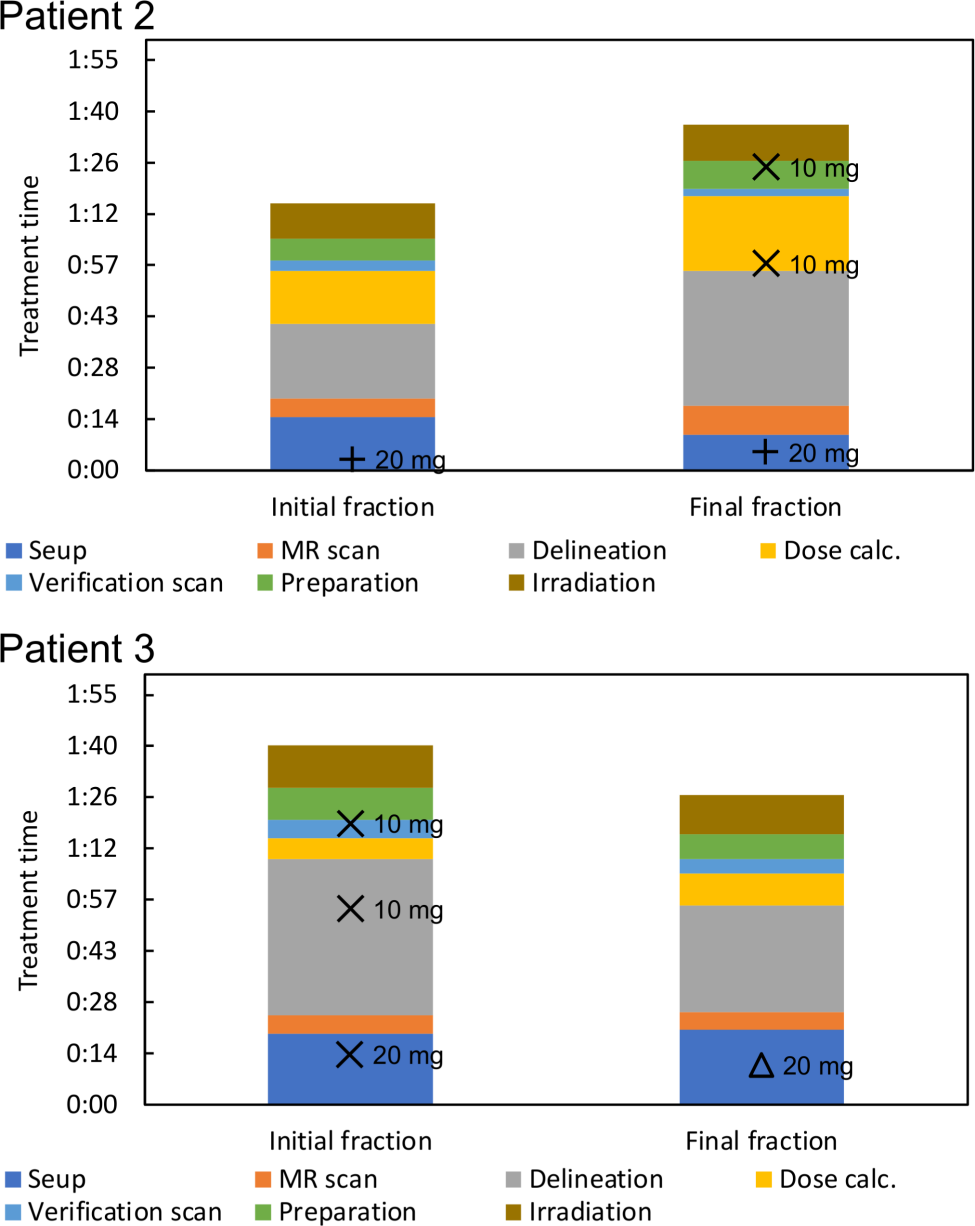
Visualization of treatment time and administration time.

Treatment was completed within 100 min for the treatment fractions included in our analysis. **Figure 3** shows the duration of treatment for each process, including administration time. Subcutaneous, intravenous, and mixed injections of butylscopolamine bromide are denoted by plus, cross, and triangular marks, respectively.

### 3.2 DSC comparison for each contour

**Figure 4:**
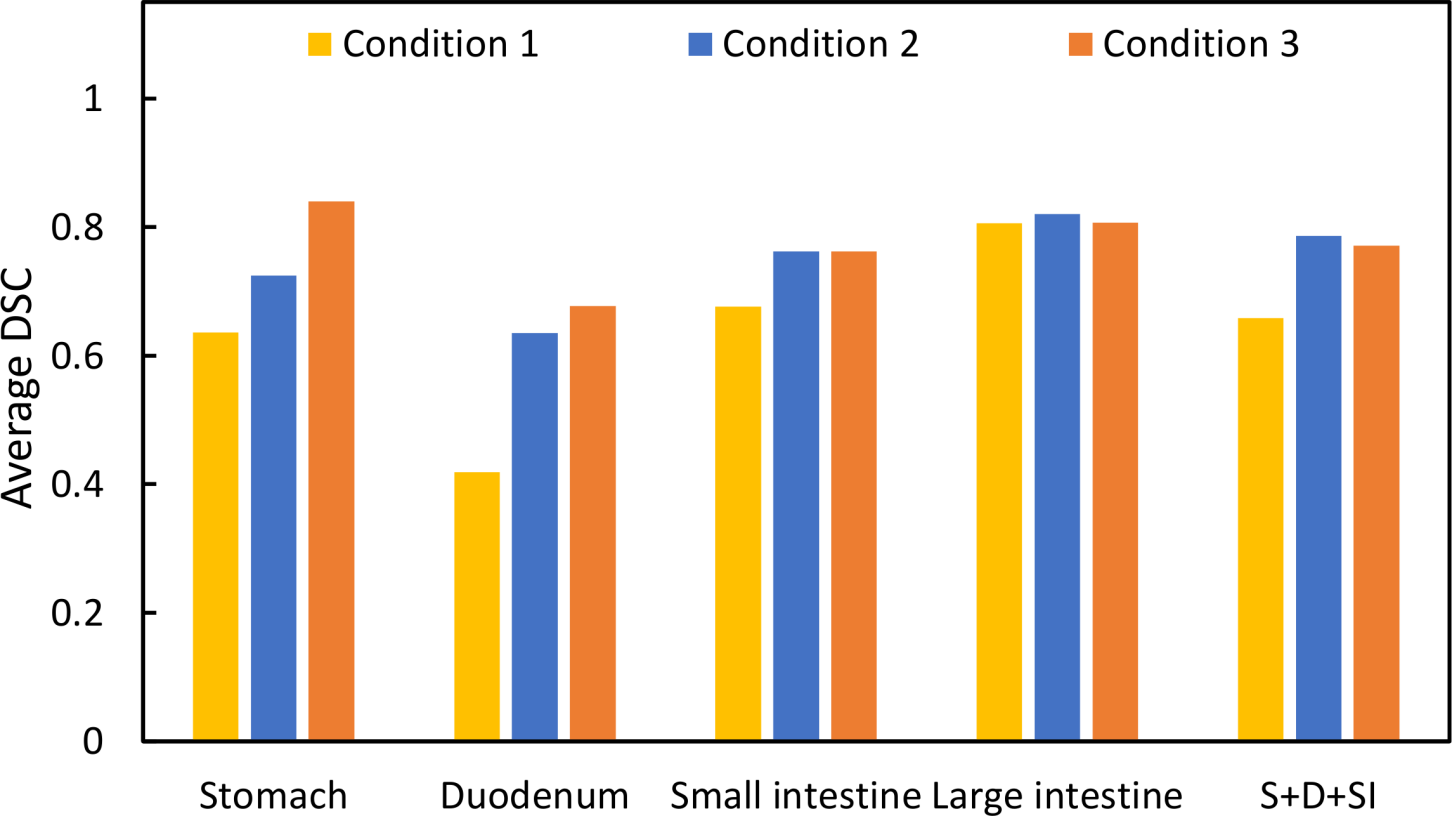
Comparison of the DSC values among the three conditions. “S+D+SI” is the outline of the stomach, duodenum, and small intestine.

**Figure 1** shows T2 Navi images of condition1 without butylscopolamine bromide administration and of conditions 2 and 3 with butylscopolamine bromide administration. The visibility of the image obtained without butylscopolamine bromide administration was poor (**Figure 1** upper row). **Figure 4** shows the DSC results for each patient. DSC values were higher for all conditions involving butylscopolamine bromide administration compared to condition 1, where butylscopolamine bromide was not administered.

### 3.3 Shortest distance and area of overlap between the PTV and OARs

**Table 3** summarizes the results of the shortest distance from the PTV to the OARs and CV for each condition, while **Table 4** lists the results of the overlap volume between the PTV and OARs and CV for each condition. The overlap volumes of the stomach and large intestine could only be calculated under one condition. Therefore, the volume of these structures was not evaluated (**Table 4**). Although SD is a measure of dispersion of population, comparing dispersion of multiple ones, it may not be an effective in analysis. In a population wherein the average value is larger, SD is likely to be larger. In such cases, the comparison takes into account the population size by dividing SD by the average value to calculate CV. In some conditions and fractions, administration resulted in lower CV values. However, no trend was found depending on whether butylscopolamine bromide was administered or not. We consider that this is because the anatomical position of OARs differs among patients and positions of OARs change between inter-fractions.

**Table 3:** Average values of the shortest distance between the planning target volume and organ-at-risk in each slice.

Average distance [mm]	Condition	Average		Coefficient of variation (↓)	
		Initial fraction	Final fraction	Initial fraction	Final fraction
Stomach	1	19.53 ± 6.67	18.56 ± 8.19	0.34	0.44
	2	N/A	N/A	N/A	N/A
	3	4.55 ± 2.96	5.70 ± 1.91	0.65	0.34
Duodenum	1	12.41 ± 3.02	2.52 ± 1.03	0.24	0.41
	2	13.44 ± 0.98	14.59 ± 5.30	0.07	0.36
	3	N/A	N/A	N/A	N/A
Small intestine	1	9.03 ± 2.77	11.10 ± 5.36	0.31	0.48
	2	26.20 ± 6.65	28.90 ± 3.90	0.25	0.14
	3	12.01 ± 4.00	24.97 ± 4.08	0.33	0.16
Large intestine	1	34.50 ± 7.74	23.24 ± 0.70	0.22	0.03
	2	62.33 ± 3.52	30.53 ± 3.55	0.06	0.12
	3	26.24 ± 1.89	33.48 ± 2.87	0.08	0.08

“N/A” indicates that the OAR does not exist on all evaluated slices and the distance is not calculated.

**Table 4:** Area of overlap between the planning target volume and organ-at-risk.

Area [cc]	Condition	Average		Coefficient of variation (↓)	
		Initial fraction	Final fraction	Initial fraction	Final fraction
Duodenum	1	1.04 ± 0.91	0.77 ± 0.43	0.87	0.56
	2	1.05 ± 0.53	0.66 ± 0.48	0.50	0.72
	3	16.85 ± 2.94	11.34 ± 0.55	0.17	0.05
Small intestine	1	6.12 ± 4.34	0.02 ± 0.03	0.71	1.34
	2	1.66 ± 2.96	N/A	1.78	N/A
	3	1.89 ± 3.05	0.07 ± 0.07	1.62	1.04

“N/A” indicates that the PTV and OAR do not overlap and the volume cannot be calculated.

### 3.4 Peristaltic motion

**Figure 5:**
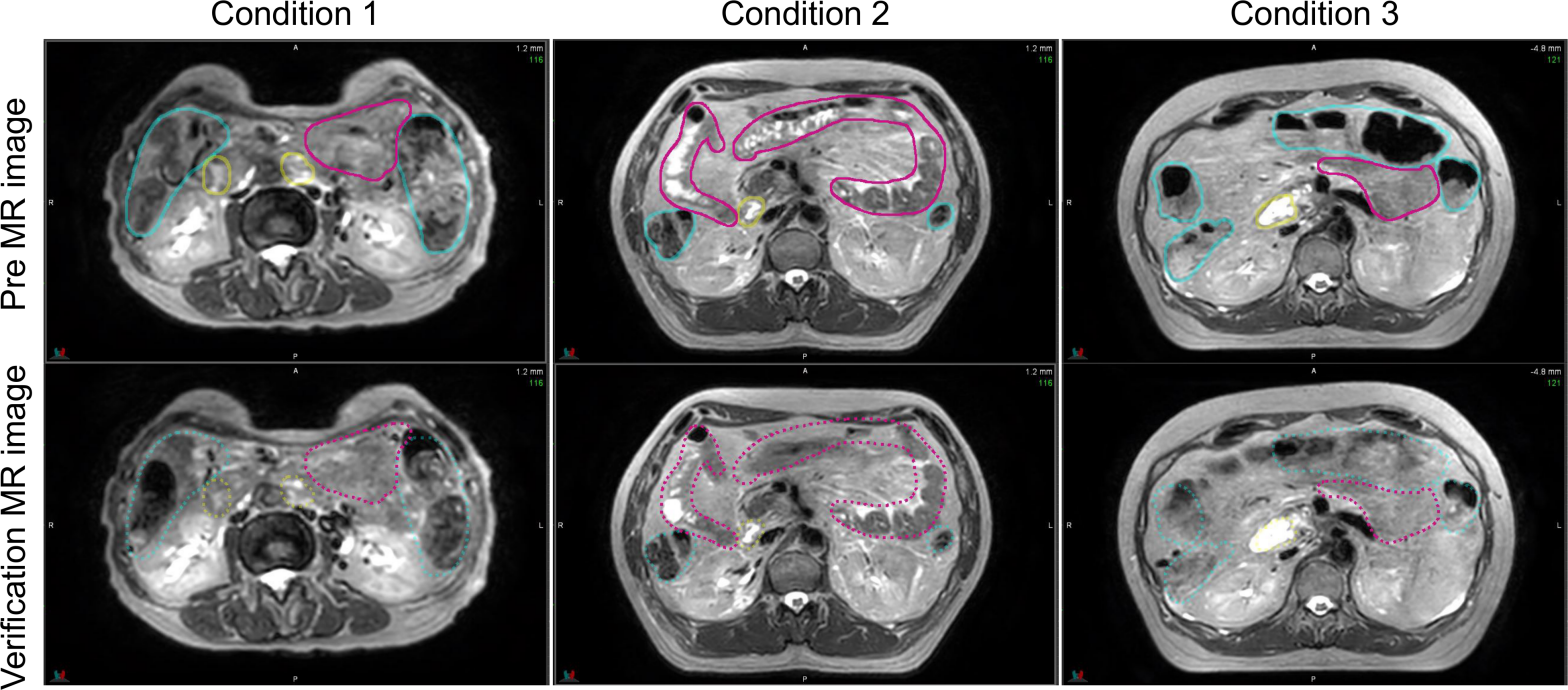
Comparison of the pre- and verification MR images for conditions 1, 2, and 3. The contour created in the pre-image is depicted on both MR images. MR, magnetic resonance.

**Figure 5** shows the pre-treatment, and position verification MR images for conditions 1, 2, and 3. The outline created by observer RO1 in the pre-treatment image is depicted in both MR images. It is a solid line on the pre-treatment MR image and a dotted line on the position verification MR image. Furthermore, yellow lines indicate the duodenum, pink lines indicate the small intestine, and light blue lines indicate the large intestine. In the conditions with butylscopolamine, although the movement of gas and water within the intestinal tract was observed, there were no major positional changes. Conversely, positional fluctuations were observed in condition 1. **Table 5** shows the results of the DSC. For all OARs, conditions 2 and 3 had higher DSC values than condition 1. Therefore, it can be inferred that butylscopolamine helps suppress peristalsis during the OART workflow.

**Table 5:** DSCs for organ-at-risk delineations extracted from pre-treatment and verification magnetic resonance imaging.

		Condition No		
		1	2	3
DSC	Stomach	0.86	0.97	0.94
	Duodenum	0.78	0.94	0.86
	Small intestine	0.88	0.90	0.89
	Large intestine	0.87	0.94	0.91

DSC, dice similarity coefficient.

## 4 Discussion

In our study, we evaluated three patients with pancreatic cancer who underwent treatment with MRgOART. Normal organ contours were visualized for each observer on MR images acquired during both the initial and final MRgOART sessions. These patients were categorized into three conditions based on the MRI sequence and butylscopolamine administration. We examined the contour delineation accuracy and investigated the conditions suitable for contour delineation. The DSC was used to verify the accuracy of contour delineation among observers and to evaluate peristaltic motion.

Evaluating DSC using the same threshold for multiple organs due to its sensitivity to contour delineation volume is challenging. Therefore, we compared the results for conditions 1, 2, and 3 within the same OAR. The average DSC value was higher in conditions wherein butylscopolamine was administered compared with those wherein it was not used. This suggests that butylscopolamine administration could contribute to the improved accuracy of OARs contour delineation. Thus, using butylscopolamine may be effective in reducing interobserver error.

Regarding the MRgOART workflow, contours are drawn using MRI. There is a concern that artifacts due to respiratory movement or peristalsis may occur during imaging. These movements may reduce the image quality and make identifying lesion challenging. Heerkens et al. evaluated respiratory migration in patients with pancreatic cancer. Respiratory migration of tumor was verified on sagittal and coronal MRI using in-house software. Their results indicated that the body moves by an average of 15 mm in the craniocaudal direction and that the deep expiration (end-expiration position) phase is the most stable (10). In this study, we adopted the T2 navigator echo imaging method, which acquires images in time with deep exhalation, which helps mitigate the effects of respiratory movement. Additionally, abdominal compression suppresses the amount of movement. Mostafaei et al. (11) concluded that peristalsis must also be assessed in addition to respiratory movement. Therefore, we used the DSC in this study to determine whether the contour changed between the pre-treatment and verification MR images to evaluate peristalsis. We found that the DSC was high when butylscopolamine was used.

Wagner et al. reported that butylscopolamine administration before MRI improves the diagnostic accuracy of lesions in the pancreatic head and body during interobserver evaluation (17). In their study, patients received an intramuscular injection of 40 mg of butylscopolamine immediately before undergoing MR imaging. It was reported that repeat imaging after 29 min (mean) did not result in a significant deterioration in image quality. MR imaging of the abdominal region following butylscopolamine administration are of significantly superior quality (18, 19). Additionally, Martí-Bonmatí et al. demonstrated the effectiveness of suppressing peristalsis using medication for MR imaging of pancreatic cancer (12). Our results also could support previous findings; hence we conclude that butylscopolamine contributes to improved contouring accuracy and reduces intra-fractional motion.

The limitations of this study are that it was conducted within a single institution; the number of conditions and observers was small. Due to the small number of cases, discussing the duration of effectiveness of butylscopolamine might be impossible. However, the evaluation of DSC between the pre- and verification-MR images confirmed the suppression of peristaltic movement in the presence of drugs. Our assessment was limited to two specific periods during the MRgOART process –before treatment planning and before irradiation– and we did not extensively evaluate intra-fractional motion. Therefore, we suggest that the following two studies will be needed: 1) an examination of interobserver error with a large number of cases and 2) an evaluation of peristaltic motion during treatment. The number of cases and observers will be increased to assure our result of interobserver error. An analysis of cine 2D MR images (5 frames/second) acquired during irradiation will be required to evaluate the effect of peristaltic suppression.

## 5 Conclusion

We used DSC to evaluate the interobserver errors in OAR delineation in patients with pancreatic cancer treated with MRgOART at our hospital. Using butylscopolamine resulted in high DSC values in all organs, suggesting that it reduces interobserver error. Furthermore, it helped reduce intra-fractional motion, in addition to improving the accuracy of contour delineation.

## Data availability statement

The raw data supporting the conclusions of this article will be made available by the authors, without undue reservation.

## Ethics statement

The studies involving humans were approved by Chiba University Clinical Research Center, Chiba University Hospital. The studies were conducted in accordance with the local legislation and institutional requirements. Written informed consent for participation was not required from the participants or the participants’ legal guardians/next of kin in accordance with the national legislation and institutional requirements.

## Author contributions

MK: Writing – original draft, Conceptualization, Funding acquisition, Project administration, Visualization. MT: Conceptualization, Data curation, Formal analysis, Project administration, Software, Visualization, Writing – original draft. KA: Formal analysis, Software, Writing – review & editing. YI: Resources, Software, Writing – review & editing. AKa: Resources, Writing – review & editing. MS: Resources, Writing – review & editing. AKo: Resources, Writing – review & editing. RH: Writing – review & editing. HY: Writing – review & editing. MW: Writing – review & editing. TU: Writing – review & editing.
